# Delivering Cognitive Behavior Therapy to Young Adults With Symptoms of Depression and Anxiety Using a Fully Automated Conversational Agent (Woebot): A Randomized Controlled Trial

**DOI:** 10.2196/mental.7785

**Published:** 2017-06-06

**Authors:** Kathleen Kara Fitzpatrick, Alison Darcy, Molly Vierhile

**Affiliations:** ^1^ Stanford School of Medicine Department of Psychiatry and Behavioral Sciences Stanford, CA United States; ^2^ Woebot Labs Inc. San Francisco, CA United States

**Keywords:** conversational agents, mobile mental health, mental health, chatbots, depression, anxiety, college students, digital health

## Abstract

**Background:**

Web-based cognitive-behavioral therapeutic (CBT) apps have demonstrated efficacy but are characterized by poor adherence. Conversational agents may offer a convenient, engaging way of getting support at any time.

**Objective:**

The objective of the study was to determine the feasibility, acceptability, and preliminary efficacy of a fully automated conversational agent to deliver a self-help program for college students who self-identify as having symptoms of anxiety and depression.

**Methods:**

In an unblinded trial, 70 individuals age 18-28 years were recruited online from a university community social media site and were randomized to receive either 2 weeks (up to 20 sessions) of self-help content derived from CBT principles in a conversational format with a text-based conversational agent (Woebot) (n=34) or were directed to the National Institute of Mental Health ebook, “Depression in College Students,” as an information-only control group (n=36). All participants completed Web-based versions of the 9-item Patient Health Questionnaire (PHQ-9), the 7-item Generalized Anxiety Disorder scale (GAD-7), and the Positive and Negative Affect Scale at baseline and 2-3 weeks later (T2).

**Results:**

Participants were on average 22.2 years old (SD 2.33), 67% female (47/70), mostly non-Hispanic (93%, 54/58), and Caucasian (79%, 46/58). Participants in the Woebot group engaged with the conversational agent an average of 12.14 (SD 2.23) times over the study period. No significant differences existed between the groups at baseline, and 83% (58/70) of participants provided data at T2 (17% attrition). Intent-to-treat univariate analysis of covariance revealed a significant group difference on depression such that those in the Woebot group significantly reduced their symptoms of depression over the study period as measured by the PHQ-9 (F=6.47; *P*=.01) while those in the information control group did not. In an analysis of completers, participants in both groups significantly reduced anxiety as measured by the GAD-7 (F_1,54_= 9.24; *P*=.004). Participants’ comments suggest that process factors were more influential on their acceptability of the program than content factors mirroring traditional therapy.

**Conclusions:**

Conversational agents appear to be a feasible, engaging, and effective way to deliver CBT.

## Introduction

Up to 74% of mental health diagnoses have their first onset before the age of 24 [1]. Depression and anxiety symptoms are particularly common among college students, with more than half reporting symptoms of anxiety and depression in the previous year that were so severe they had difficulty functioning [2]. In addition, epidemiological data suggest that mental health problems are both increasing in prevalence and severity [3]. However, up to 75% of the college students that need them do not access clinical services [3]. While the reasons for this are varied, the ubiquity of free or inexpensive mental health services on campuses suggests that service availability and cost are not primary barriers to care [3]. Like non-college populations, stigma is considered the primary barrier to accessing psychological health services.

Overcoming problems of stigma has been traditionally considered a major benefit of Internet-delivered and more recently mobile mental health interventions. In recent years, there has been an explosion of interest and development of such services to either supplement existing mental health treatments or expand limited access to quality mental health services [4]. This development is matched by great patient demand with about 70% showing interest in using mobile apps to self-monitor and self-manage their mental health [5]. Internet interventions for anxiety and depression have empirical support [6] with outcomes comparable to therapist-delivered cognitive behavioral therapy (CBT) [7,8]. Yet, despite demonstrated efficacy, they are characterized by relatively poor adoption and adherence. One review found a median minimal completion rate of 56% [9]. A hypothesized reason for this lack of adherence is the loss of the human interactional quality that in-person CBT retains. For example, certain therapeutic process factors such as accountability may be more salient in traditional face-to-face treatments, compared to digital health interventions.

With recent advancements in voice recognition, conversational interfaces (ie, those that use natural language as inputs and outputs) have begun to emerge. Conversational agents (such as Apple’s Siri or Amazon’s Alexa) may be a more natural medium through which individuals engage with technology. Humans respond and converse with nonhuman agents in ways that mirror emotional and social discourse dynamics when discussing behavioral health [10] and their capacity to act as first responders has already been evaluated [11]. Theoretically, conversational interfaces may be better positioned than visually oriented mobile apps to deliver structured, manualized therapies because in addition to delivering therapeutic content, they can mirror therapeutic process. Indeed, Bickmore et al demonstrated that a carefully designed health-related conversational agent could establish a therapeutic relationship with adults attempting to increase exercise [10]. The intervention was an embodied conversational agent, that is, it was designed with a graphical face to mirror human interactions that are typically face-to-face.

However, most consumer-facing conversational agents are not embodied. The capacity of text-based agents to deliver CBT is a question worth exploring given the ability of widely disseminated evidence-based digital apps to reduce the burden of mental illnesses in the US college population, estimated to be approximately 20 million [12]. Unfortunately, the few mobile apps that have been evaluated formally have seen substantial challenges to sustainability since they tend to be built in academic research settings and rarely have the required infrastructure to support them. One systematic review of 5464 abstracts identified just 5 apps that had supporting evidence from randomized controlled trials, though, as of January 2014, none of them were available commercially [13]. Thus, in the interest of sustainability, this study tested the ability of a commercially developed text-based conversational agent to deliver CBT to college students.

Given the variability in quality of available mental health apps, a conversational agent was created to integrate 15 out of the 16 evidence-based recommendations for app development [4] as follows: built using a CBT framework; addressing both anxiety and low mood; designed for use by nonclinical populations; incorporating automated tailoring; reporting of thoughts, feelings, or behaviors; recommending activities; provision of mental health information; real-time engagement; activities explicitly linked to specific reported mood problems; encouraging non‒technology-based activities; gamification and intrinsic motivation to engage; reminders to engage; simple and intuitive interface and interactions; and including links to crisis support services. While these recommendations were created in the context of mobile phone apps, to our knowledge, their relevance in the context of a conversational interface has never been tested.

Thus, the objective of this study was to assess the feasibility of delivering CBT in a conversational interface via an automated bot in a way that facilitates engagement and reduction in symptoms. The current study compared outcomes from 2 weeks of a CBT-oriented conversational agent (Woebot), or an information control group (National Institute of Mental Health’s [NIMH] ebook) in a nonclinical college population. We hypothesized that conversation with a therapeutic process-oriented conversational agent would lead to greater improvement in symptoms relative to the information control group. We also hypothesized that receiving psychoeducational material in a conversational manner would be more acceptable to those who received it.

## Methods

### Recruitment and Procedure

Potential participants were recruited using a flyer posted on social media websites targeting a US university community for students who self-identified as experiencing symptoms of depression and anxiety. Inclusion criteria included age 18 and over (screened at the first level via checkbox confirmation) and able to read English (implied). To guard against compromise, for example from malicious bots, all potential participants were sent an email requesting that they respond denoting their confirmation. Confirmed participants were randomized via computer algorithm that automatically generated a number between 0 and 1. Participants with numbers ˂0.5 were allocated to receive a direct link to begin chatting with Woebot in an instant messenger app, and participants with numbers >0.5 were sent a link to NIMH’s ebook on depression among college students [14], after completion of online baseline questionnaires. Because the randomization allocation occurred algorithmically, allocation concealment was in place. However, the condition to which each participant was allocated was not masked for the service providers (Woebot Labs). After approximately 2 weeks (T2), participants were contacted again to complete a second set of questionnaires online. Participants were offered a prorated incentive of US $10 per completed assessment (US $20 for completion of both assessments).

Since this trial involved a nonclinical population of college students, it was considered exempt from registration in a public trials registry. See Multimedia Appendix 1 for the study’s CONSORT-EHEALTH checklist [15].

### Interventions

#### Woebot

Woebot is an automated conversational agent designed to deliver CBT in the format of brief, daily conversations and mood tracking. Woebot is used within an instant messenger app that is platform agnostic and can be used either on a desktop or mobile device. Each interaction begins with a general inquiry about context (eg, “What’s going on in your world right now?”), and mood (eg, “How are you feeling?”) with responses provided as word or emoji images to represent affect in that moment. After gathering mood data, participants are presented with core concepts related to CBT by link to short video, or by way of short “word games” designed to facilitate teaching participants about cognitive distortions. The first day included an “onboarding” process that introduced the bot, adding that while the bot may seem like a person, it is closer to a “choose your own adventure self-help book” and therefore not fully capable of understanding what the needs of the user may be. The bot also briefly explained CBT and notified the user that while a psychologist was “keeping an eye on things” (ie, monitoring), this was not happening in real time and thus the service should not be used as a replacement for therapy. In addition, participants were encouraged to call 911 for emergencies.

The bot employed several computational methods depending on the specific section or feature. The overarching methodology was a decision tree with suggested responses that also accepted natural language inputs with discrete sections of natural language processing techniques embedded at specific points in the tree to determine routing to subsequent conversational nodes. For the duration of the study, the decision tree structure remained the same for each participant and parameters did not change depending on the participants’ inputs. Weekly graphs were processed using temporal pattern recognition to provide users with weekly mood description.

The bot’s conversational style was modeled on human clinical decision making and the dynamics of social discourse. Psychoeducational content was adapted from self-help for CBT [16-18]. Aside from CBT content, the bot was created to include the following therapeutic process-oriented features:

Empathic responses: The bot replied in an empathic way appropriate to the participants’ inputted mood. For example, in response to endorsed loneliness, it replied “I’m so sorry you’re feeling lonely. I guess we all feel a little lonely sometimes” or it showed excitement, “Yay, always good to hear that!”

Tailoring: Specific content is sent to individuals depending on mood state. For example, a participant indicating that they feel anxious is offered in-vivo assistance with the anxious event.

Goal setting: The conversational agent asked participants if they had a personal goal that they hoped to achieve over the 2-week period.

Accountability: To facilitate a sense of accountability, the bot set expectations of regular check-ins and followed up on earlier activities, for example, on the status of the stated goal.

Motivation and engagement: To engage the individual in daily monitoring, the bot sent one personalized message every day or every other day to initiate a conversation (ie, prompting). In addition, “emojis” and animated gifs with messages that provide positive reinforcement were used to encourage effort and completion of tasks.

Reflection: The bot also provided weekly charts depicting each participant’s mood over time. Each graph was sent with a brief description of the data to facilitate reflection, for example, “Overall, your mood has been fairly steady, though you tend to become tired after periods of anxiety. It looks like Tuesday was your best day.”

#### Information Control Condition

In the information control condition, participants were directed to the NIMH resources section and specifically, a free publication entitled “Depression in College Students” [14]. The ebook provides comprehensive evidence-based information on depression among college students including sections on signs and symptoms, different types of treatments, answers to frequently asked questions, and a list of resources including further reading, helpline numbers, and other resources.

### Measures

#### The Patient Health Questionnaire-9

The Patient Health Questionnaire (PHQ-9) [19] is a 9-item, self-report questionnaire that assesses the frequency and severity of depressive symptomatology within the previous 2 weeks. It is one of the most widely used, reliable, and validated measures of depressive symptoms. Each of the 9 items is based on the *Diagnostic and Statistical Manual of Mental Disorders*, 4^th^ edition (DSM-IV) criteria for major depressive disorder and can be scored on a 0 (not at all) to 3 (nearly every day) scale. Scores ranging from 0-5 indicate no symptoms of depression, and scores of 5-9, 10-14, 15-20, and ˃20 representing mild, moderate, moderately severe, and severe depression, respectively.

#### Generalized Anxiety Disorder-7

The Generalized Anxiety Disorder 7-item scale (GAD-7) [20] is a valid, brief self-report tool to assess the frequency and severity of anxious thoughts and behaviors over the past 2 weeks. Based on the DSM-IV diagnostic criteria for GAD, the scores of all 7 items range from 0 (not at all) to 3 (nearly every day). Therefore, the total score ranges from 0-21. A score ˃10 is indicative of moderate anxiety, with a score greater than 15 indicating severe anxiety.

#### Positive and Negative Affect Schedule

The Positive and Negative Affect Schedule (PANAS) [21] is a 20-item self-report measure of current positive and negative affect. Half the items represent positive affect (ie, interested, excited, determined), whereas half of the items are indicative of negative affect (ie, hostile, scared, ashamed). Items are scored on a 1 (very slightly or not at all) to 5 (extremely) scale, with higher scores representing higher affect. Positive and negative affect are summed independent of each other with possible scores from 10-50.

#### Acceptability and Usability

Mixed-format questions assessed feasibility and acceptability of both conditions. Participants from both groups were asked to rate on a 5-point Likert scale their level of overall satisfaction and satisfaction with content (0=hated it, 5=loved it, 3=neutral, 2 and 4 unlabeled); the extent to which they felt the intervention facilitated emotional awareness (0=not at all, 5=a lot, 3=neutral, 2 and 4 unlabeled); whether or not they learned anything (binary, yes/no response), and to what extent this learning was relevant to their everyday life (0=not at all, 5=a lot, 3=neutral, 2 and 4 unlabeled). In addition, participants were asked what the best and worst thing about their experience was and to provide other comments. While we were mainly interested in qualitative responses pertaining to the Woebot condition, responses to the information control allowed for an informal assessment of engagement. Finally, for those in the Woebot condition, we recorded total number of interactions (ie, conversations) with the bot over the 2-week period. An interaction was deemed to have taken place if mood and context data were recorded. Session or conversation length varied from approximately 90 seconds to 10 minutes, depending on psychoeducational content.

### Statistical Analysis

Statistical power calculations using analysis of covariance (ANCOVA) revealed that a sample size of 70 would have sufficient (80%) power to detect a moderate-large effect size (Cohen *d*=0.4) for depression, reported by a meta-analysis of Internet-delivered treatments for adult depression and anxiety [8], with alpha at 5%.

To determine whether any significant differences between groups existed at baseline, independent *t* tests were conducted on continuous baseline variables (eg, age, PHQ-9, GAD-7, and PANAS), and chi-square analyses were conducted on categorical or nominal variables (gender, race, ethnicity). Univariate effects of group membership on T2 outcomes were examined using between-subjects ANCOVA adjusting for baseline measures. Cohen *d* effect sizes were calculated to examine the magnitude of between-group differences. All subjects were included in intention-to-treat (ITT) analyses. Prior to conducting these analyses, the multiple imputation procedure in SPSS v. 23 was used to handle missing data assumed to be missing at random.

As secondary subgroup analyses, we conducted completer analyses using 2x2 repeated measures analysis of variance (ANOVA) to explore main and interaction effects.

#### Qualitative Analysis

Participants’ responses to open-ended questions were analyzed for the Woebot group using only thematic analysis and were reported as frequencies. Data were analyzed thematically using an inductive (data-driven) approach guided by the procedure outlined by Braun and Clarke [22]. Data codes were generated systematically, then collated into “thematic maps” and applied to the entire dataset to generate frequencies.

### Ethics and Informed Consent

The study was reviewed and approved by Stanford School of Medicine’s Institutional Review Board. Participants indicated their consent to the terms of the study via checkbox on an information sheet. As additional safety measures, participants in the Woebot group who denoted long-standing depression, suicidality, or self-harm were automatically provided with helpline numbers and a crisis text line number, and were encouraged to call 911 in emergencies.

With the exception of data on usage, which were collected by the Life Ninja Project, all study data were collected by the academic institution. Because of deidentification of all data transmitted between the Life Ninja Project and Stanford, usage data were not linked to specific research participants and are reported as means only for the entire group of study participants.

## Results

**Figure 1:**
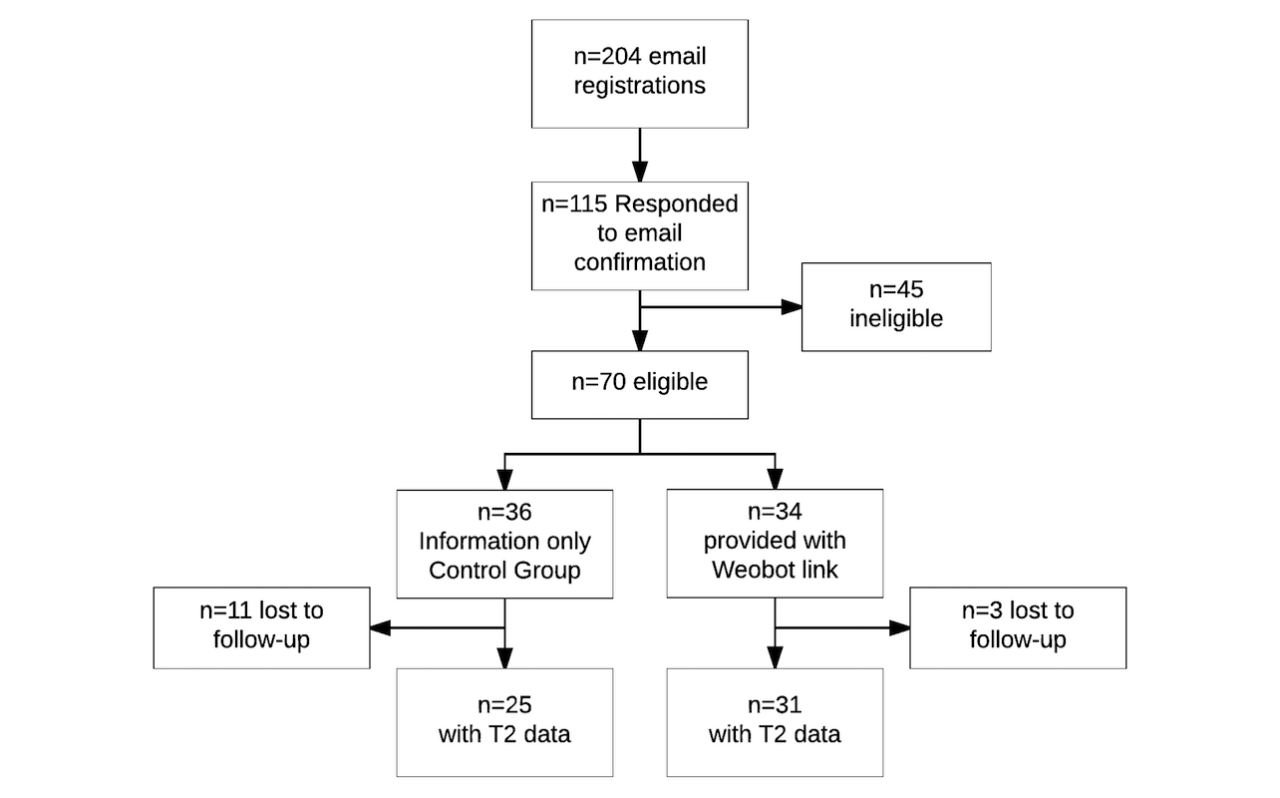
Participant recruitment flow.

Figure 1 shows the participant flow throughout the study. A total of 204 registrations were received between January 31 and February 20, 2017, and all registrants were asked to confirm their interest by return email. A total of 115 responded to this email, though 45 of these were deemed bot-generated (eg, email addresses with unusual almost identical formats and identical responses) and were deemed ineligible. The resultant sample of N=70 were randomized via computer algorithm to receive either a direct link to begin chatting with Woebot (n=34) in an instant messenger app, or NIMH’s ebook on depression among college students [14] (n=36), after completion of online questionnaires at baseline.

### Attrition

Of the randomized participants, 83% (58/70) went on to provide partial or complete data at T2 representing an overall attrition rate of 17%. Attrition was not equal between the arms and was greater among the information control group (31% vs 9%; χ^2^_1_=5.16; *P*=.023). However, independent *t* tests and chi-square analyses failed to detect evidence of significant differences at baseline between those who dropped out of the study versus those who did not on age (*t*_68_=1.18; *P*=.24); GAD-7 (*t*_68_=1.28; *P*=.89); PHQ-9 (*t*_68_=.63; *P*=.59); PANAS positive (*t*_68_=.79; *P*=.43) and negative (*t*_68_=.02; *P*=.98) affect scores; or on gender (χ^2^_1_=1.75; *P*=.18) or ethnicity (χ^2^_1_=.066; *P*=.79).

### Participant Demographics

Table 1 shows the demographic information and baseline scores on clinical variables for those with data from the entire sample (N=58). Participants were an average of 22.2 years old (SD 2.33) and over two-thirds female. Participants were mostly non-Hispanic (93%, 54/58), 79% Caucasian (46/58), with 7% (4/58) Asian, 9% (5/58) more than one race, 2% (2/58) African American, and 2% (2/58) Native American/Alaskan Native.

In terms of baseline characteristics, nearly half (46%, 32/69) of the sample was in the moderately-severe or severe range of depression at baseline as measured by the PHQ-9, while three-quarters (74%, 52/70) were in the severe range for anxiety as measured by the GAD-7.

**Table 1:** Demographic and clinical variables of participants at baseline.

		Information control	Woebot
**Scale, mean (SD)**			
	Depression (PHQ-9)	13.25 (5.17)	14.30 (6.65)
	Anxiety (GAD-7)	19.02 (4.27)	18.05 (5.89)
	Positive affect	26.19 (8.37)	25.54 (9.58)
	Negative affect	28.74 (8.92)	24.87 (8.13)
Age, mean (SD)		21.83 (2.24)	22.58 (2.38)
**Gender, n (%)**			
	Male	4 (7)	7 (21)
	Female	20 (55)	27 (79)
**Ethnicity, n (%)**			
	Latino/Hispanic	2 (8)	2 (6)
	Non-Latino/Hispanic	22 (92)	32 (94)
	Caucasian	18 (75)	28 (82)
	Non-Caucasian	6 (25)	6 (18)

**Table 2:** Results of ITT analysis of entire sample on primary outcomes in the study at T2.

	Information-only control		Woebot		*F*	*P*	*d*^c^
	T2^a^	95% CI^b^	T2^a^	95% CI^b^			
PHQ-9	13.67 (.81)	12.07-15.27	11.14 (0.71)	9.74-12.32	6.03	.017	0.44
GAD-7	16.84 (.67)	15.52-18.56	17.35 (0.60)	16.16-18.13	0.38	.581	0.14
PANAS positive affect	26.02 (1.45)	23.17-28.86	26.88 (1.29)	24.35-29.41	0.17	.707	0.02
PANAS negative affect	27.53 (1.42)	24.73-30.32	25.98 (1.24)	23.54-28.42	0.91	.912	0.344

^a^Baseline=pooled mean (standard error)

^b^95% confidence interval.

^c^Cohen *d* shown for between-subjects effects using means and standard errors at Time 2.

**Figure 2:**
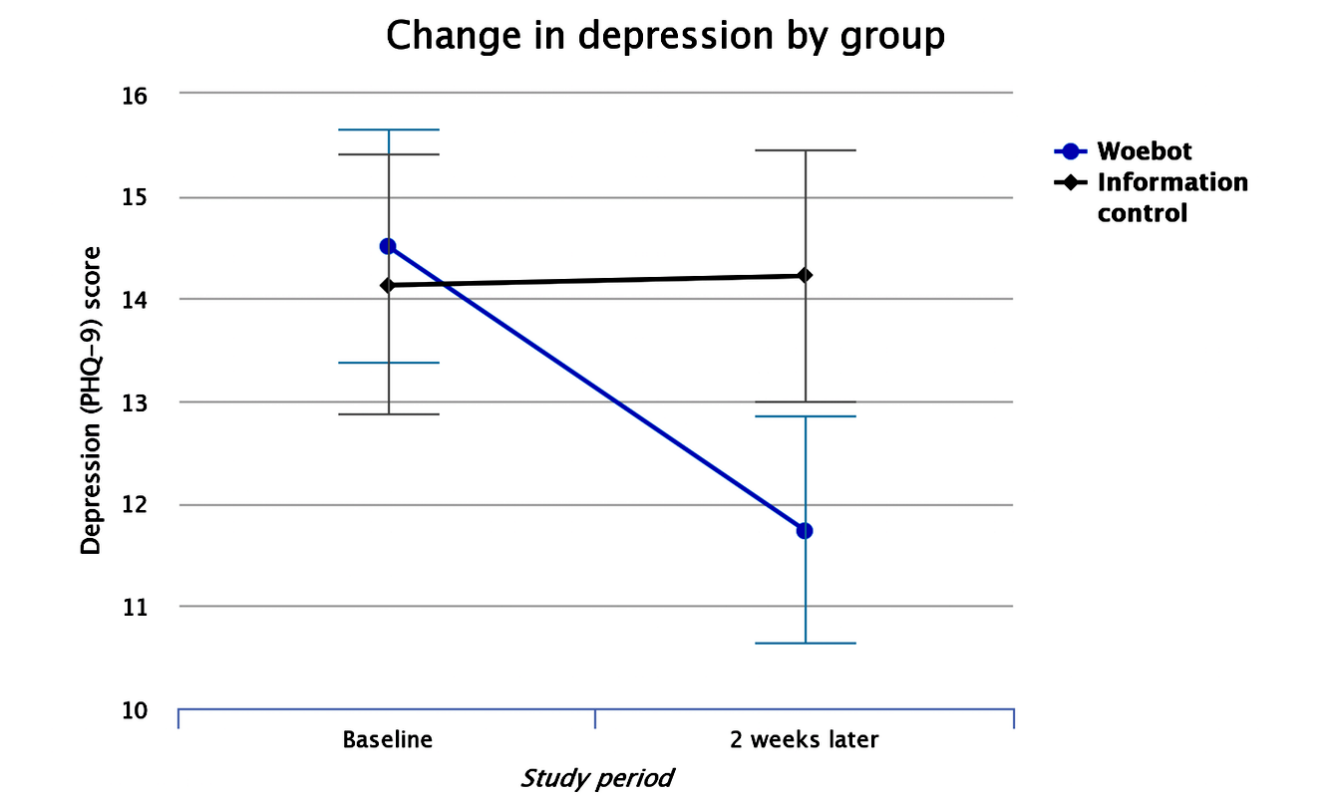
Change in mean depression (PHQ-9) score by group over the study period. Error bars represent standard error.

### Preliminary Efficacy

Table 2 shows the results of the primary ITT analyses conducted on the entire sample. Univariate ANCOVA revealed a significant treatment effect on depression revealing that those in the Woebot group significantly reduced PHQ-9 score while those in the information control group did not (*F*_1,48_=6.03; *P*=.017) (see Figure 2). This represented a moderate between-groups effect size (*d*=0.44). This effect is robust after Bonferroni correction for multiple comparisons (*P*=.04). No other significant between-group differences were observed on anxiety or affect.

### Completer Analysis

As a secondary analysis, to explore whether any main effects existed, 2x2 repeated measures ANOVAs were conducted on the primary outcome variables (with the exception of PHQ-9) among completers only. A significant main effect was observed on GAD-7 (*F*_1,54_=9.24; *P*=.004) suggesting that completers experienced a significant reduction in symptoms of anxiety between baseline and T2, regardless of the group to which they were assigned with a within-subjects effect size of *d*=0.37. No main effects were observed for positive (*F*_1,50_=.001; *P*=.951; *d*=0.21) or negative affect (*F*_1,50_=.06; *P*=.80; *d*=0.003) as measured by the PANAS.

To further elucidate the source and magnitude of change in depression, repeated measures dependent *t* tests were conducted and Cohen *d* effect sizes were calculated on individual items of the PHQ-9 among those in the Woebot condition. The analysis revealed that baseline-T2 changes were observed on the following items in order of decreasing magnitude: motoric symptoms (*d*=2.09), appetite (*d*=0.65), little interest or pleasure in things (*d*=0.44), feeling bad about self (*d*=0.40), and concentration (*d*=0.39), and suicidal thoughts (*d*=0.30), feeling down (*d*=0.14), sleep (*d*=0.12), and energy (*d*=0.06).

### Use and Acceptability

Participants in the Woebot condition checked in with the bot (defined as at least providing context and mood information) an average of 12.14 times (SD 2.23; median 12; range 8-18) over the 2-week period, with almost all check-ins occurring on unique days. Since we could not track website visits, page views, click-through rates, etc, of NIMH’s website that hosted the ebook, we have no means of confirming to what extent individuals in the information control group engaged with the material. However, a total of 13 (52%) provided detailed comments suggesting they had read the ebook at least once.

While ratings indicated that both conditions were acceptable (above 3/5), participants in the Woebot condition reported significantly higher levels of satisfaction both overall (4.3 versus 3.4; *t*_48_=3.99; *P*<.001) and with content (4.0 versus 3.4; *t*_48_=2.30; *P*=.02), and they reported a significantly greater amount of emotional awareness as a result of using the bot (3.3 versus 2.7; *t*_47.06_=2.38; *P*=.021) than the information control group. All (100%) of the participants in the Woebot group endorsed having learned something new versus three-quarters (77%) of the information control group, though numbers were too small in some cells to allow for a chi-square analysis. There was no difference between groups in how relevant participants viewed that learning to everyday life.

### Qualitative Results

**Figure 3:**
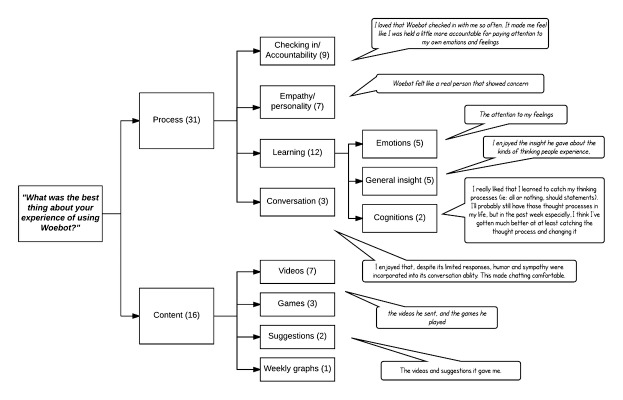
Thematic map of participants’ most favored features of their experience of using Woebot.

Figure 3 shows a thematic map of participants’ responses to the question “What was the best thing about your experience using Woebot?” Two major themes emerged in respect to this question: process and content. In the process theme, the subthemes that emerged were accountability from daily check-ins (noted by 9 participants); the empathy that the bot showed, or other factors relating to his “personality” (n=7); and the learning that the bot facilitated (n=12), which in turn was divided into further subthemes of emotional insight (n=5), general insight (n=5), and insights about cognitions (n=2).

**Figure 4:**
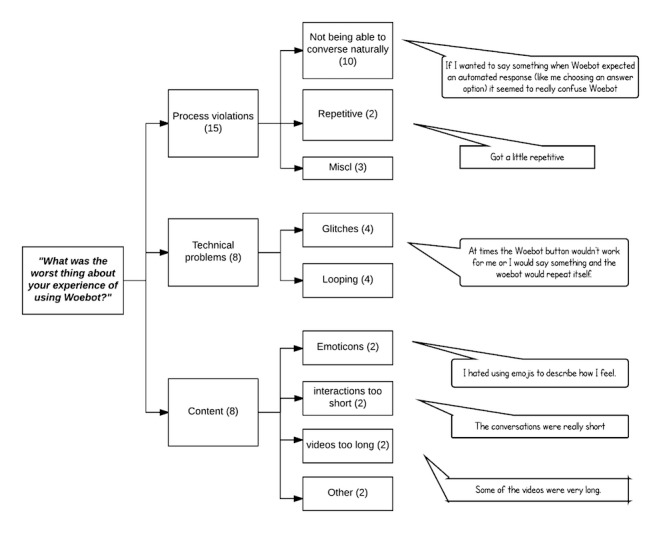
Thematic map of participants’ least favored experiences using Woebot.

Figure 4 illustrates a thematic map of participants’ responses to the question: “What was the worst thing about your experience with Woebot?” Three themes emerged: process violations (n=15), technical problems (n=8), and problems with content (n=8). By far the most common subtheme to emerge among the process violations related to the limitations in natural conversation such as the bot not being able to understand some responses or getting confused when unexpected answers were provided by participants (n=10), and 2 individuals noted that the conversations could get repetitive. Technical problems were described by 8 individuals, with technical glitches in general (n=4) and looping conversational segments (n=4) emerging as subthemes. Problems with content were described by 8 individuals, with most of these relating to emoticons and either interactions or content length.

A total of 11 “other comments” were received, which were all positive, either expressing gratitude for the experience: “I love Woebot so much. I hope we can be friends forever. I actually feel super good and happy when I see that it ‘remembered’ to check in with me!” Statements described how helpful it was: “I really was impressed and surprised at the difference the bot made in my everyday life in terms of noticing the types of thinking I was having and changing it”. Many spoke about Woebot in interpersonal terms, for example, “Woebot is a fun little dude and I hope he continues improving.”

## Discussion

### Principal Results

To our knowledge this is the first randomized trial of a nonembodied text-based conversational agent designed for therapeutic use. The objective of the study was to explore whether a fully automated conversational agent based on CBT principals could deliver a therapeutic experience to college students over a 2-week period. We hypothesized that a conversational agent built to incorporate both evidence-based guidelines for the development of mental health apps as well as hypothesized therapeutic process variables would be highly engaging, more acceptable, and would lead to greater reductions in symptoms of anxiety and depression relative to an information control group.

The study confirmed that after 2 weeks, those in the Woebot group experienced a significant reduction in depression, thus our hypothesis was partially supported. Woebot was associated with a high level of engagement with most individuals using the bot nearly every day and was generally viewed more favorably than the information-only comparison.

### Comparisons With Prior Work

Using Woebot was associated with a significant reduction in depression as measured by the PHQ-9. The effect size for depression was moderate though smaller than the four published studies [23-26] that describe three other mobile app interventions targeting depression. For example, Burns et al [26] found a reduction in depression symptoms with a between-groups effect size of 1.9, and Watts et al also found significant reductions in PHQ-9 scores with an effect size of 1.56, both after an 8-week program. However, these interventions were much longer in duration than Woebot, which was just 2 weeks long. Indeed, our effect size for reduction in depression is in line with that observed in a randomized trial of DBT Coach [27], a mobile app for individuals with borderline personality disorder, who received a similar dose of 14 days.

The number of participants reporting that the bot felt empathic is noteworthy, and comments that referred to the bot as “he,” “a friend,” and a “fun little dude” suggest that the perceived source of empathy was Woebot rather than the bot’s developers. This is especially noteworthy since a purposefully robotic name “Woebot” was chosen to emphasize the nonhuman nature of the agent. This is in line with other work that suggests that therapeutic relationship can be established between humans and nonhuman agents in the context of health and mental health. For example, Bickmore et al [10] have demonstrated that individuals using a bot to encourage physical activity developed a measurable therapeutic bond with the conversational agent after 30 days. This embodied bot was built on substantial design work on establishing human-computer relationships [28]. In addition, a trial that compared therapeutic engagement with a nonhuman agent between individuals randomized to think that there was a human operating the agent or not demonstrated that individuals were more willing to disclose to an artificially intelligent “virtual therapist” than when they believed it was human-operated [29]. The results of this preliminary trial suggest that this should be explored explicitly in future studies, ideally employing a standardized measure of working alliance, such as the Working Alliance Inventory [30].

The frequency of process-related comments made by participants in response to questions about their experience with Woebot suggests that conversational agents can approximate some therapeutic process factors. In addition, just as these factors are thought to convey much of the variance in positive outcomes across therapeutic approaches, this study suggests that conversational agent process factors, such as the ability to convey empathy, may be capable of both amplifying and conversely, violating, a therapeutic process. This underscores the importance of including trained and seasoned clinicians in clinical app design processes. While this point has been suggested, for example in the recent guidelines for clinical app evaluation published by the American Psychiatric Association [31], and in the United Kingdom by the National Institute for Health and Care Excellence [32], this study goes some way towards illustrating the impact that therapeutic process variables may have on user experience in the context of mental health apps.

### Limitations

There are several methodological weaknesses that limit the generalizability of the findings. As a feasibility study, we recruited a limited number of participants to receive a relatively short intervention, and no follow-up data were available to assess whether gains were sustained. The small number of participants meant that a formal mediator analysis was not possible, thus we cannot formally test a theorized relationship between engagement and outcome in this context of conversational agents. The study should be replicated with more participants, a longer dose, and a follow-up period to investigate if findings persist. In addition, sufficient numbers to test for mediation effects would inform theory. Aside from indirectly inferring from comments, objective quantitative data on engagement were not available for the information-only control group, thus it was not possible to compare engagement between the two groups in a meaningful way. In addition, because data were deidentified, it was not possible to explore whether any dose-response effects existed. Nonetheless, the relatively strong comparison group can be viewed as a strength of the study. Indeed, the relative strength of the control group was illustrated by the fact that individuals providing data in that group saw a similar reduction in anxiety as those who received Woebot, which supports the literature that suggests minimal passive psychoeducation alone can reduce symptoms of psychological distress [33]. Nonetheless, the choice of control group was somewhat limiting for two main reasons. First, it may have contributed to the high attrition rate since an ebook is not designed for multiple or recurring sessions. It also did not introduce any CBT-specific material, thus it was not possible to evaluate whether the conversational delivery mediated symptom reduction, rather than the CBT content that the bot delivered. In order to answer this question adequately, future research should incorporate an interactive online CBT self-help intervention as a comparison condition.

Finally, the study was conducted in a New York area university community population and since we did not formally assess digital divide factors such as socioeconomic status, findings may be limited in their generalizability.

### Conclusions

While results should be viewed with some caution and the findings need to be replicated, this study nonetheless demonstrates that a text-based conversational agent designed to mirror therapeutic process has the potential to offer an alternative and engaging method of delivering CBT for some 10 million college students in the United States who experience debilitating anxiety and depression.

## Appendix

Multimedia Appendix 1 CONSORT-EHEALTH checklist V1.6.2.

## Abbreviations

ANCOVA: analysis of covariance
ANOVA: analysis of variance
DSM-IV: Diagnostic and Statistical Manual of Mental Disorders, 4th edition
GAD-7: Generalized Anxiety Disorders scale
ITT: intention to treat
NIMH: National Institute of Mental Health
PANAS: Positive and Negative Affect Scale
PHQ-9: Patient Health Questionnaire scale
T2: time 2
